# Microbial links to Alzheimer’s disease

**DOI:** 10.1371/journal.ppat.1013599

**Published:** 2025-10-23

**Authors:** Courtney P. Cornitius, Emily K. Perez, Soo Chan Lee

**Affiliations:** South Texas Center for Emerging Infectious Diseases (STCEID), Department of Molecular Microbiology and Immunology, The University of Texas at San Antonio, San Antonio, Texas, United States of America; Tufts Univ School of Medicine, UNITED STATES OF AMERICA

## Abstract

Alzheimer’s disease (AD) is a common chronic neurodegenerative disease that is characterized by (1) plaques, (2) neuronal death, (3) hyperphosphorylation of tau proteins, and (4) memory deficits. Despite extensive research, the underlying cause of AD is not fully understood. Evidence that infectious pathogens may trigger AD has been documented for decades. There are notable correlations between microbes (including bacteria, viruses, and fungi) and AD pathologies. Although it is yet to be determined if pathogenic microbes are the causative agent or a contributing factor to AD, the role of infections in the pathogenesis of AD should not be ignored. Even though the pathogens may not cause AD, they may play a direct role in the triggering or exacerbation of AD progression. In this mini review, the current status of pathogens’ role in AD etiology will be presented.

## Introduction

Alzheimer’s disease (AD) is currently ranked as the seventh leading cause of death in the United States and is the most common cause of dementia. In the United States alone, this disorder affects at least two-thirds of dementia patients [1]. Approximately 1 in 9 individuals aged 65 and above (10.8%) are diagnosed with AD, with 1,275 new cases occurring annually per 100,000 in the United States alone [2]. A large majority of AD cases are categorized as sporadic, while a small subgroup (~ 5%) has an early onset of the disease. Early onset (EOAD) is defined as having an age less than 65 years at the time of onset and is linked to autosomal dominant mutations of three genes: β-amyloid precursor protein (APP), presenilin 1 (PSEN1), and presenilin 2 (PSEN2). In contrast, the more common late onset (LOAD) is primarily associated with the genetic risk factor of ε4 allele of apolipoprotein E (Apo ε4) [3,4]. AD is characterized as a neurodegenerative disorder with symptoms including memory loss, cognitive impairments, and behavioral changes. Postmortem pathological features include extracellular deposits of amyloid-β (Aβ) plaques, intracellular neurofibrillary tangles of phosphorylated tau protein, and neuronal death and loss [5]. AD causality is multifactorial; main risk factors are associated with age [6], genetic predisposition [7], traumatic brain injury [8], and numerous environmental factors [9]. Despite this knowledge, these risk factors fail to explain the systemic neuroinflammation associated with AD patients; alternative hypotheses need to be investigated. Usually, AD is thought to be correlated with the appearance of Aβ plaques, resulting in hyperphosphorylation of the tau protein, which in turn leads to neuronal death. With this line of thought, clinical trials were aimed at decreasing the presence of Aβ plaques. The latest class of medication available for AD patients only aims to mitigate symptoms with the hopes of slowing disease progression. While traditionally understood as a primarily genetic and age-related condition, emerging research suggests a complex interplay between bacterial interactions and disease progression. The microbiota–gut–brain axis (MGBA) may play roles in the development and progression of AD. Dysbiosis in the microbiome in the gut is known to be linked to elevated permeability of the GI barrier, resulting in inflammation and neurodegeneration [10]. The disruptions of the gut barrier may enable microbes in the GI tract and/or their effectors to migrate into the brain [10]. There is growing evidence supporting that Aβ-plaques may have a beneficial role(s) in protecting the body from infections [11]. More specifically, Aβ-peptides have antimicrobial properties, suggesting pathogens can induce plaque formation and AD may have a microbial origin [12]. Most research in this field focuses on individual pathogens [13] rather than the hypothesis of polymicrobial causality, despite growing support for this hypothesis [14–16]. The recognition of the theory that AD may have a microbial origin may open the doors to novel approaches in diagnosis, treatment, and prevention. The focus of this review is to highlight the mounting evidence demonstrating the potential role bacteria, fungi, and viruses play in the contributions to the onset and/or the progression of AD.

## Bacteria

**Fig 1:**
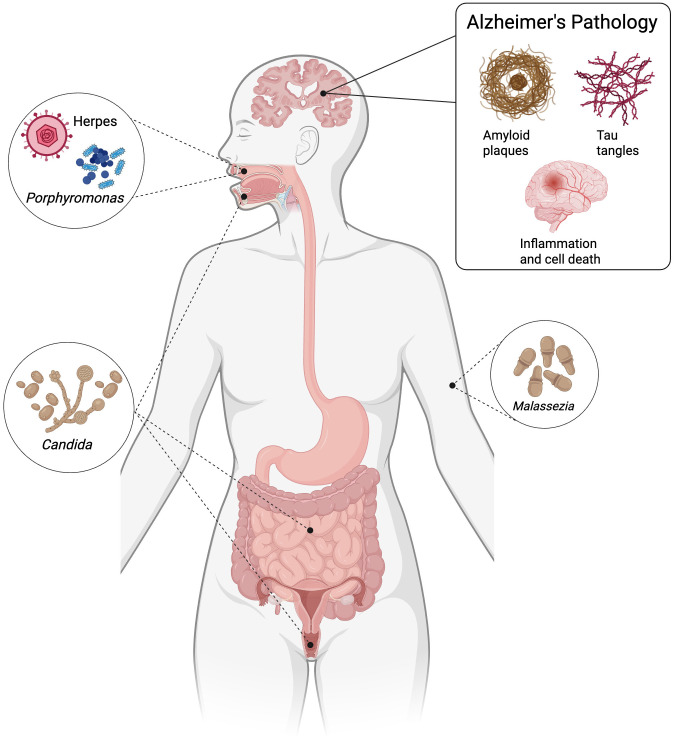
Contributions of pathogens to the pathogenesis of Alzheimer’s disease (AD). Pathogens are involved in the onset and or progression of AD results in one or more pathological markers: (1) amyloid-β deposit, (2) increased tau phosphorylation, (3) neuroinflammation. Created with BioRender.com.

Recent scientific investigations have uncovered intriguing connections between bacterial presence and Alzheimer’s pathology, supporting decades of discussion on the possibility that microorganisms may play a role in the disease’s etiology. One of the first documented evidence of bacteria contributing to the development of neurological disorders belonged to the phylum *Spirochaetes* [17,18]. Specific bacterial pathogens include *Chlamydia pneumoniae*, a cause of lung infections, several types of spirochaete bacterium like *Borrelia burgdorferi*, the agent of Lyme disease, and, most recently, *Porphyromonas gingivalis*, which leads to gum disease, have been identified as potential contributors to neurological damage (Fig 1) [19]. The studies have proposed that chronic bacterial infections might contribute to neuroinflammation and amyloid-β protein accumulation, hallmark features of AD [20]. Oral bacteria represent a significant area of research. A recent study demonstrated that *P. gingivalis*, a pathogen associated with chronic periodontitis, can invade brain tissue and potentially trigger neuroinflammatory responses. The bacterial enzyme gingipain was found to correlate with increased damage and amyloid-β formation [21]. Another study outlines that *P. gingivalis* exposure alone significantly increased neuronal damage, activation of astrocytes and microglia, increased expression of the inflammatory cytokines interleukin-1β (IL-1β) and interleukin-6 (IL-6), and the production of amyloid-β plaques and hyperphosphorylated tau in the hippocampus, cortex, and midbrain [22]. *P. gingivalis* can promote neuroinflammation and injury via several mechanisms, such as the gut-brain axis, thereby impairing neuronal growth and survival and playing a role in the development and progression of neurodegenerative disorders [21–23]. Additionally, it has been reported that *P. gingivalis* in animal models can induce neuroinflammation by activating the Toll-like receptor 4/nuclear factor-kappa B signaling pathway [24], cause cognitive impairment by secreting pro-inflammatory cytokines [25], and lead to brain colonization and accumulation of Aβ [21,26]. The gut-brain axis is a critical pathway, suggesting that bacterial populations could influence neurological health through complex immunological mechanisms.

Moreover, research on the microbiome has greatly expanded our understanding of the role bacteria may play in neurodegenerative diseases. Researchers have revealed that the gut microbiome of AD participants has decreased microbial diversity. Investigators have explored how systemic bacterial infections and chronic inflammation might accelerate neurodegeneration [27]. The microbiome of AD participants was noted to have a decrease in *Firmicutes* and *Bifidobacterium* and an increase in *Bacteroidetes*. In bacterial genera that are more abundant in AD, there was an observed relationship between increased bacterial abundance and greater AD pathology [27]. This pathology included a greater burden of β-amyloid plaques, elevated levels of phosphorylated tau in the brain, and increased concentrations of chitinase-3-like protein 1 [27].

A finding such as this strongly suggests a potential connection between gut health and the development of AD pathology. It implies that disruptions in the gut microbiome, or the ability of pathogens to influence systemic inflammation and neuroinflammatory pathways, may play a critical role in initiating or accelerating the neurodegenerative processes characteristic of AD. Developing evidence indicates that bacterial presence might not merely be a consequence of neurodegeneration but potentially a causative agent. Chronic bacterial infections could trigger inflammatory responses, compromise neuronal integrity, and accelerate cognitive decline.

## Fungi

Fungal cell components have been detected in both post-mortem brain tissue and cerebrospinal fluid (CSF) samples from individuals diagnosed with AD [28,29]. This is particularly noteworthy given the absence of such fungal elements in non-AD control samples. Researchers have found multiple fungal species, such as *Candida*, *Malassezia*, and others, in AD samples. While these species have been detected, no single species has been consistently found across all brain regions [29,30]. The increased presence of fungal bodies in AD patients suggests a potential link between fungal infections and AD pathology. As a result, the possible role of fungi in neurodegenerative disease has become an emerging focus in recent AD research, with growing interest in how the immune system’s response to fungal pathogens may contribute to disease progression. Additionally, the presence of fungi in AD brains begs the question, where are the fungi coming from? Are they migrating from other regions of the host, such as the oral cavity, gastrointestinal tract, or vulvovaginal tract?

One fungal species of interest is *Candida albicans*, which is capable of hematogenous dissemination, crossing the blood–brain barrier (BBB) through both paracellular and transcellular mechanisms. These include direct invasion of endothelial cells and the exploitation of host immune cells, particularly monocytes and macrophages, as “Trojan horses” to gain access to the brain parenchyma [31–33]. *C. albicans* has been shown to enter the mouse brain from the bloodstream and activate two key neuroimmune sensing pathways. These involve secreted aspartic proteinases (Saps) and the fungal toxin candidalysin. Saps disrupt the tight junctions of the BBB, facilitating fungal dissemination and invasion of brain tissue [34]. They also cleave APP into Aβ-like peptides, which bind to Toll-like receptor 4 (TLR4) and promote fungal clearance [34]. Candidalysin, on the other hand, activates microglia through the integrin receptor CD11b, further driving the immune response [34]. In a low-grade *C. albicans* infection mouse model, microglia and astrocytes are observed to cluster around yeast cells, releasing pro-inflammatory cytokines such as IL-1β, IL-6, and TNF to help eliminate the fungus, and more importantly, the fungus causes AD-like symptoms [35]. In addition, this immune activity mirrors inflammatory responses seen in AD, where microglia surround damaged neurons, amyloid-β plaques, and hyperphosphorylated tau, releasing the same cytokines [36]. While this inflammatory response may initially serve a protective function, its chronic activation leads to progressive neuronal damage—a hallmark of AD. Notably, the loss of candidalysin recognition via CD11b significantly prolongs *C. albicans* brain infections, highlighting the importance of this pathway in fungal clearance [34].

Together, these findings suggest that the innate immune response to fungal infections, particularly *C. albicans*, shares mechanistic similarities with the neuroinflammation observed in AD (Fig 1). The overlapping pathways of inflammation and neuronal degeneration support a possible role of fungal–host interactions in AD progression, warranting further investigation into the contribution of fungal pathogens to neurodegenerative disease.

## Viruses

The relationship between viral infections and AD has emerged as a critical area of neurological research. Recent studies have revealed complex mechanisms by which viruses may contribute to neurodegeneration, challenging traditional knowledge of the disease’s etiology. One line of thought about the development of AD is centered around the reactivation of the latent herpes virus. *Herpesviridae* is a family of double-stranded DNA viruses, eight of which are known to infect humans and cause neurological disease: herpes simplex-1 (HSV-1), herpes simplex-2 (HSV-2), cytomegalovirus (CMV), Epstein-Barr virus (EBV), varicella-zoster virus (VZV), human herpesvirus 6 (HHV-6), human herpesvirus 7 (HHV-7), human herpesvirus 8 (HHV-8), and hepatitis C virus (HCV) [37–41]. Herpetic viruses may increase inflammation, susceptibility to the development of AD, and a decrease in cognitive decline [42]. One PCR-based study demonstrated AD samples contained an increased presence of HHV-6 and HSV-1 when compared to healthy controls [40]. The *Herpesviridae* viruses might be a risk factor for AD when it occurs concomitantly with APOE ε4, a known risk factor for AD. When the virus acts with APOE ε4, it causes accumulation of Aβ, an increase in the formation of amyloid plaques, and inflammatory damage (Fig 1) [43,44]. Moreover, another study provided compelling evidence suggesting that HSV-1 infection might play a significant role in AD pathogenesis. The researchers found an association between HSV carriage and individuals with the APOE ε4 genetic variant [45–47]. Additionally, it has been previously discussed that human neuroblastoma cells infected with HSV-2 have increased accumulation of amyloid plaques and tau protein [48]. Several published studies have noted that HCV can infect monocytes, cross the BBB, and therefore trigger an immune response through the excessive secretion of TNF-α and IL-6. These cytokines then cause neuroinflammation and altered cerebral metabolism [49]. Lastly, another landmark study reported utilizing a 3D-brain organoid system using human-induced neural stem cells (hiNSCs) to replicate an AD model by introducing HSV-1 infection. Without using any external AD-inducing agents (e.g., mutant cell lines), the model replicates key disease features such as amyloid plaque structures, gliosis, neuroinflammation, and functional decline [50].

Building on this, both direct viral effects and expanded T cell responses to viral antigens have been implicated in AD pathology. Rising research has highlighted the potential impact of other viral agents. A comprehensive review explored multiple viral interactions, identifying HHV6A and 7 as potentially influential in neuroinflammatory processes [38]. EBV-specific T cells, peripheral CD8+ TEMRA cells (terminal effector memory cells re-expressing CD45RA) in particular, have been shown to contribute to neuroinflammation and cognitive decline in AD patients [51], with more recent work describing other pathogen-specific T cell expansions that may exacerbate neurodegenerative processes [52]. Additionally, SARS-CoV-2, the virus responsible for COVID-19, and influenza have researchers introducing new ideas into the viral neurological field. Studies have demonstrated potential neurological complications, suggesting that viral infections might accelerate or trigger neurodegenerative processes [53,54]. Research shows that after COVID-19 infections, especially in aged rodent models, CD8+ T cells infiltrate the brain where they become highly activated, correlating with impaired spatial learning, increased neuronal death, and reduced regeneration. These findings suggest that CD8+ T cells directly contribute to cognitive dysfunction and may help explain the neurological symptoms and long-term effects seen in conditions such as long COVID [55,56].

Altogether, these findings suggest that viral exposures and infections not only influence molecular and cellular pathways directly but also shape adaptive immune responses that may increase the risk of neurodegenerative diseases such as Alzheimer’s.

## Future directions

While an increasing body of literature supports associations between microbial infections and AD, the strength of this evidence remains largely correlative rather than causative. For instance, the presence of bacterial DNA, fungal components, or viral particles in AD brains does not prove that these pathogens directly initiate disease; they may simply reflect secondary colonization of already vulnerable tissue. Similarly, the antimicrobial properties of amyloid-β (Aβ) peptides suggest a potential immune role, but this does not necessarily mean that microbial infection is the driving factor behind plaque formation. Antimicrobial activity is a broad phenomenon, and many host proteins share such functions without being induced by infection.

Another important limitation lies in the mechanistic interpretation. For example, neuroinflammatory responses to *C. albicans* or *P. gingivalis* resemble the immune activation observed in AD, yet similarity in immune response does not imply equivalence in etiology. This type of reasoning risks conflicting correlation with causation and could lead to flawed conclusions that any pathogen capable of eliciting inflammation is a candidate driver of AD. Likewise, viral associations, such as HSV-1 in APOE ε4 carriers, are compelling but confounded by the fact that viral reactivation is common in aging populations, making it difficult to disentangle coincidence from pathogenic contribution.

The literature is also marked by variability and inconsistency. Different studies often identify different pathogens, with no single organism consistently present across all AD cases. This raises the possibility that microbial involvement may be opportunistic or secondary, rather than primary. The hypothesis of polymicrobial causality is intriguing, but it remains underresearched. Additionally, methodological challenges further complicate interpretation. Many studies rely on PCR or immunohistochemistry, which may be prone to contamination or false positives. Small sample sizes, post-mortem delays, and heterogeneous patient populations weaken causative claims.

While these findings are promising, researchers emphasize the need for comprehensive studies to definitively establish if there is a relationship between pathogen interactions and AD progression. More insight into this field may open new approaches for understanding, treating, or even preventing AD. Many studies currently focus on a primary infectious burden, yet the role of microbiome dysbiosis remains understudied. It is well established that host microbiomes contain vast communities, and altered microbial profiles have been observed in AD patients compared to healthy controls. However, how dysbiosis directly contributes to AD pathology remains unclear. Important unanswered questions include: Do AD-related genetic risk factors predispose patients to infections? Do pathogens initiate AD, or do they merely exacerbate existing pathology? Is disease progression influenced by a single pathogen or by a multi-pathogen interplay? Finally, could antimicrobial interventions alter outcomes, especially given that Aβ itself may function as an antimicrobial peptide? These questions emphasize the complexity of the microbial hypothesis and the need for further investigation.
